# Hypercalcemia of malignancy in a dog with cutaneous apocrine gland carcinoma and malignant myoepithelioma

**DOI:** 10.1007/s11259-025-11011-4

**Published:** 2026-01-20

**Authors:** Ludovica Emiliani Pescetelli, Ambra Luisa Misia, Giulia Moretti, Giovanni Angeli, Maria Teresa Antognoni, Antonello Bufalari, Eleonora Scorsi, Elvio Lepri

**Affiliations:** 1 Private practitioner, Veterinary Clinic Giaconella, Via Eugenio Checchi 57, Rome, 00157 Italy; 2 Department of Veterinary Medicine, University of Perugia, Via San Costanzo, 4, Perugia, 06126 Italy; 3 CDvet Research, Via Giovanni Nicotera, 7, Roma, 00195 Italy

**Keywords:** Dog, Hypercalcemia of malignancy, Apocrine tumor, Immunohistochemistry

## Abstract

Humoral Hypercalcemia of Malignancy (HHM) is a commonly reported condition in dogs affected by several cancers, mostly T-cell lymphoma and anal sac gland carcinomas, but also other type of tumors. We report the case of a 6-years-old, female neutered Border Collie with an ulcered nodule on the right retromandibular region, that had mild serum total calcium elevation (14,1 mg/dl; reference range (RR): 8,4 to 11 mg/dl) in the absence of clinicopathological abnormalities or imaging finding suggesting any known cause of hypercalcemia. Histologically, the tumor had a tubular epithelial component, admixed with a second, dominant, myoepithelial one; immunohistochemically, the former strongly reacted with anti-Cytokeratin antibodies, while the latter had a weak and patchy immunoreactivity both to Cytokeratin and a-SMA, while Vimentin and Calponin immunostaining was strong and diffuse, suggesting the diagnosis of apocrine gland carcinoma and malignant myoepithelioma. After the excision of the tumor, calcium decreased within limits (8.8 mg/dl RR: 8.4 to 11. mg/dl). In the author’s knowledge, this is the first report of HHM associated with apocrine sweat gland carcinoma in the dog.

**Supplementary Information:**

The online version contains supplementary material available at 10.1007/s11259-025-11011-4.

## Introduction

Hypercalcemia (commonly defined as an increase of serum total calcium (tCa) above 11.5 mg/dL and/or ionized serum calcium (iCa) above 6 mg/dL) (Schneck et al. 2012) is a common finding in canine species, and can be associated to several diseases or conditions among which renal failure (both acute and chronic), hypoadrenocorticism, primary hyperparathyroidism (hyperplasia or adenoma of parathyroid glands), vitamin D (or analogues) intoxication, excess in dietary calcium or systemic granulomatous inflammation (Elliott, 1991); however, cancer is the underlying disease in 2/3 of canine patients with hypercalcemia (Kleiter et al. 2001). Cancer-associated hypercalcemia can be the result of bone resorption during primary or secondary bone tumors, or be mediated by the production of soluble factors (namely Parathormone-related proteins: PTH-rp) by neoplastic cells (Humoral Hypercalcemia of Malignancy: HHM). The tumors most commonly associated with HHM are lymphoma (mostly T-cell lymphomas), anal sac gland carcinomas, multiple myeloma and a variety of other tumors (among which, but not exclusively, thyroid carcinoma, squamous cell carcinoma, mammary carcinoma, melanoma, primary lung tumors, nasal carcinomas, etc.) (Meuten et al. 1983).

To the best of the authors’ knowledge, hypercalcemia has never been described in association with cutaneous apocrine sweat gland tumors.

## Case presentation

A 6-years-old, female neutered Border Collie was referred to the Veterinary Teaching Hospital of the Department of Veterinary Medicine of Perugia (Italy) due to an ulcered nodule, about 4 cm in diameter, in the right retromandibular region. Upon examination, the dog was in good body condition. Ipsilateral mandibular lymph nodes were enlarged; otherwise, general physical examination was unremarkable.

A fine needle aspiration of the nodule was performed; cytological examination revealed two distinct population of cells: small, tightly cohesive, basaloid cells organized in large clusters with occasional acinar pattern (Supplementary figure 1), and larger cells, loosely arranged and associated with a bright pink extracellular material (Supplementary figure 2). The basaloid cells were polygonal in shape, about 20 µ in diameter, with scarce to moderate amount of deeply blue micro-vacuolated cytoplasm, and oval central nucleus with coarse chromatin and one to multiple prominent central nucleoli; the second population consisted in oval to elongated cells with cytoplasmic tails, embedded in deeply pink extracellular material; the cells have moderately abundant pale blue cytoplasm and oval nucleus with reticular chromatin and inconspicuous nucleoli. Both populations exhibited prominent anisocytosis and anisokaryosis; no mitotic figures were seen. The cytological diagnosis was of a biphasic epithelial, probably complex, tumor with some criteria of malignancy.

**Fig. 1:**
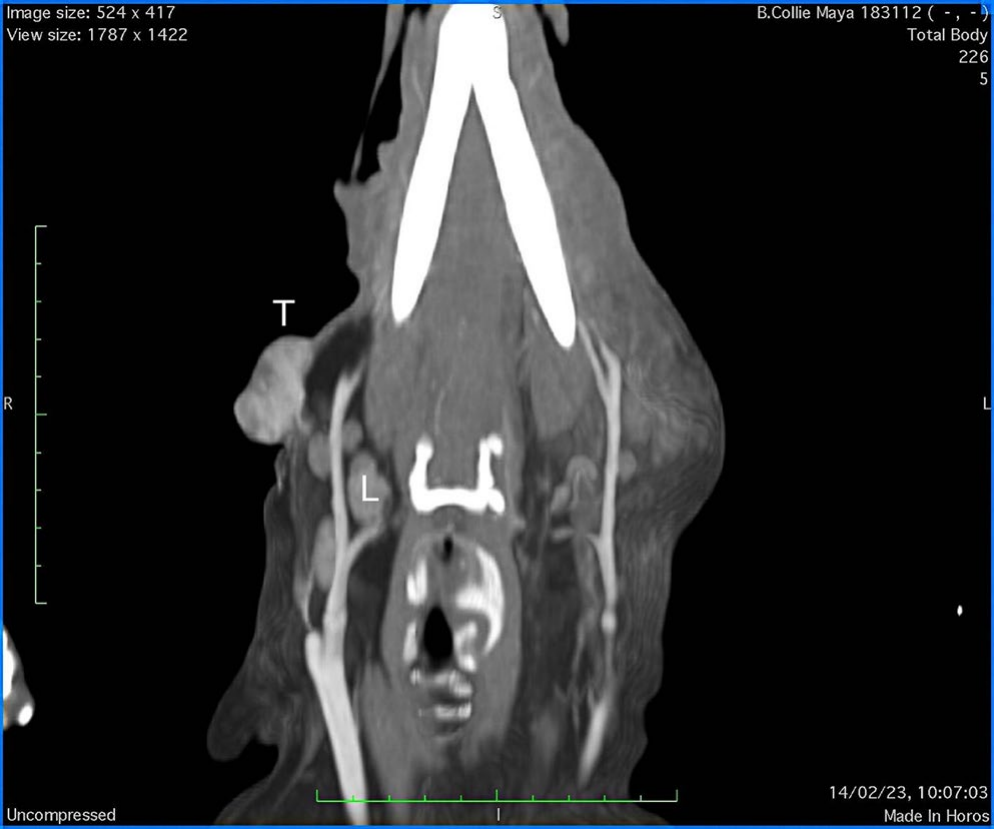
CT scan of the head of the dog; T: tumor, L: lymph-nodes

The preanesthetic complete blood count (CBC) and serum biochemistry panel were within the reference ranges, except for serum calcium (14,1 mg/dl; reference range (RR): 8,4 to 11 mg/dl). Blood Urea Nitrogen (BUN) and creatinine were normal, phosphorus within the range even to upper limit (5.1 mg/dl; reference range (RR): 2.5–5.2 mg/ml); additionally, Na/K ratio was 28,84. A thorough examination of perianal region was unremarkable. The dog underwent echocardiographic, thoracic X-ray and ultrasound abdominal examination, all of which were normal. A total-body Computed Tomography was performed as a pre-surgical screening in order to evaluate the presence of metastatic lesions. On CT scan the tumor extended from the skin and subcutaneous tissue of right retro-mandibular region and was strongly enhanced by contrast medium with an internal multilobulate hypoattenuating pseudo-cystic pattern; ipsilateral mandibular lymph nodes were enlarged and showed a homogeneous contrast enhancement (Fig. 1). Furthermore, a wide subcutaneous mass with tomographic features similar to adipose tissue, consistent with lipoma, was reported in the right gluteal region. No anomalies in other organ or signs of metastatic diffusion were found.

**Fig. 2:**
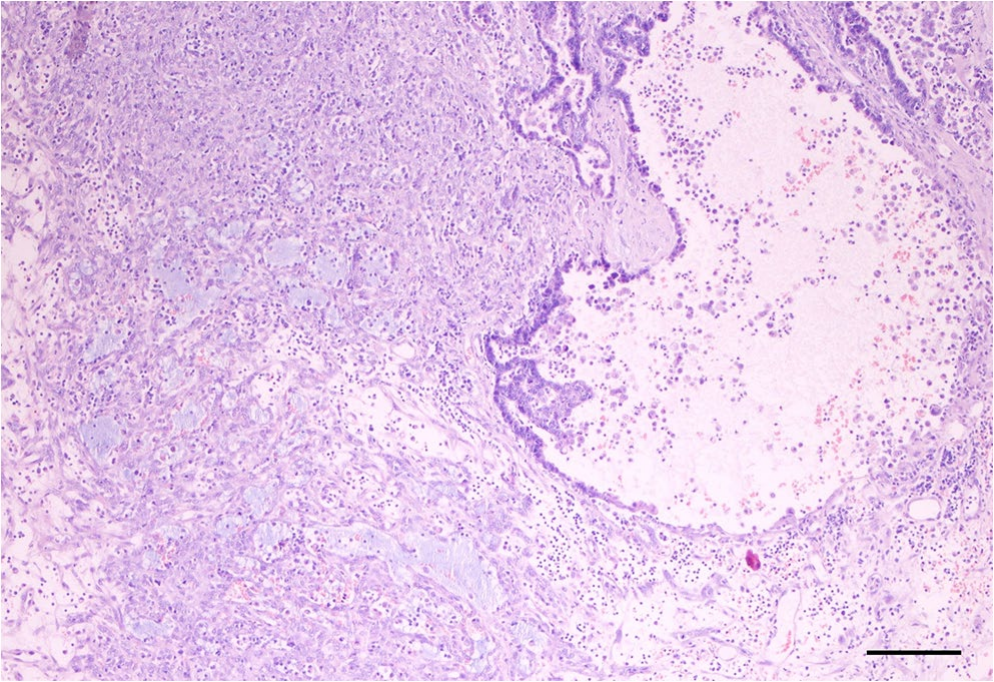
Skin, dog: the tumor has a biphasic appearance with dilated tubular structures (upper right) and a more prominent myoepithelial component with abundant basophilic extracellular myxoid stroma (lower left). HE, bar 100 μm

The cutaneous nodule and three mandibular lymph nodes were surgically removed under general anesthesia. Intraoperative lymphangiography was performed to identify sentinel lymph nodes. A 0.4 ml bolus of methylene blue was injected peritumorally just prior surgery: lymphangiography identified only one of the three retromandibular lymph nodes with a marked blue staining while the other two lymph nodes were colorless. The tumor was then excised with 2 cm of lateral margin and one fascial plane deep, with a subsequent linear pattern of surgical reconstruction. The excised tumor was formalin-fixed and routinely processed (dehydrated in alcohol, clarified in xylene and embedded in paraffin wax); 6 μm slides were stained with Hematoxylin and Eosin (HE). Histologically, the nodule was composed by a well-demarcated unencapsulated tumor with expansile growth, arising from superficial dermis and extending to the muscle layer. The tumor was composed of epithelial tubules, accounting for 20–30% of the population, lined by monolayered cuboidal cells with scant well-defined cytoplasm and rare cytoplasmic apical blebs; the nuclei were irregular and oval with prominent central nucleoli. The remaining 70–80% of the neoplasm was composed by cells ranging from polygonal to spindle-shaped, with a moderate amount of cytoplasm and oval nuclei with single or multiple non-prominent nucleoli, embedded in a loose myxoid stroma with multifocal mucin lakes containing macrophages (Fig. 2).

Anisocytosis and anisokaryosis were prominent in the tubular component, and moderate in the stromal one. Mitotic activity was 32 in 2.37 mm² with several atypical mitoses in both epithelial and mesenchymal-like components. Additionally, there are multifocal confluent necrotic areas involving approximately 30% of the tumor. Intratumoral lymphocytic infiltration and peritumoral lymphoplasmacytic infiltration were also present. (Supplementary figures 3–4)

The lymph nodes show diffuse hyperplasia with cortical follicular hyperplasia and sinus histiocytosis.

**Fig. 3:**
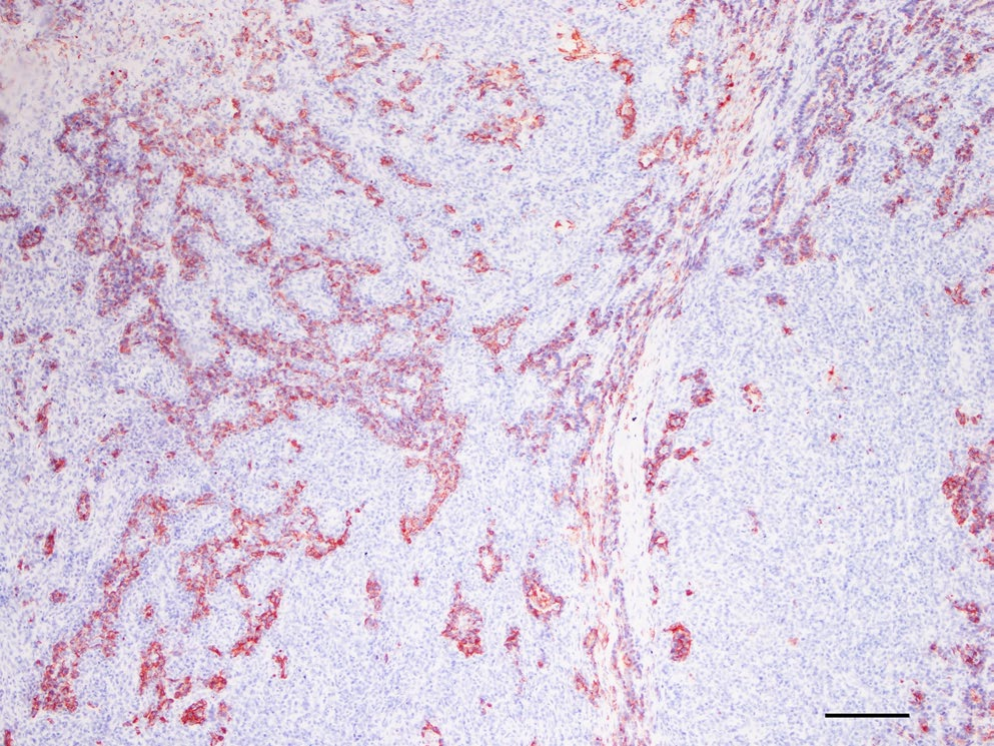
Skin, dog. Complementary images of the same microscopic field immunostained with Cytokeratin (Fig. 3), bar 200µ

**Fig. 4:**
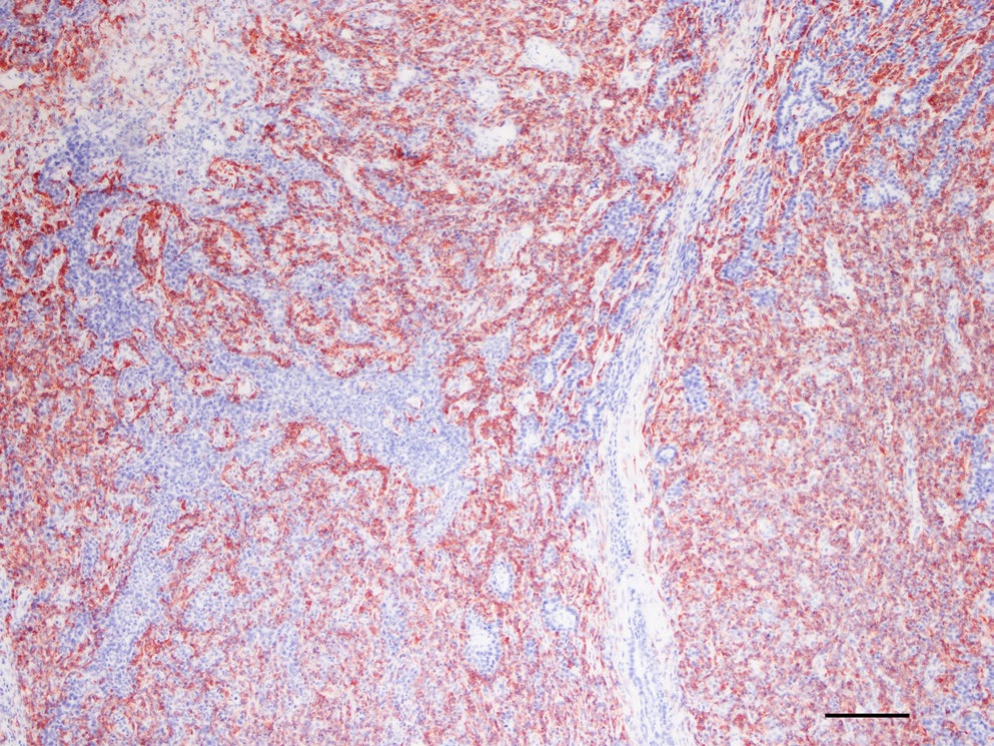
Complementary images of the same microscopic field immunostained with Calponin (Fig. 4), bar 200µ

Immunohistochemistry was performed as follows; briefly, the slides were dewaxed in xylene and rehydrated in alcohol; antigen retrieval was performed in microwave in TBS pH 6 (a-SMA) or 9 (Cytokeratin, Vimentin and Calponin); primary antibodies consisted in anti-panCytokeratin (monoclonal mouse AE1/AE3, 1:200, Dako), anti-Vimentin (monoclonal mouse V9, 1:200, Dako), anti-α-Smooth Muscle Actin (α-SMA) (monoclonal mouse IA4, 1:200, Dako) and anti-Calponin (monoclonal rabbit EP798Y, 1:1000, Abcam); a secondary antibody conjugated with Streptavidin-Biotin (BYOTINYLATED LINK, Abcam) was applied as in manufacturer instructions, followed by aminoethyl carbazole (AEC) as a chromogen. Negative controls were run by omitting the primary antibody. Internal positive controls were represented by epidermis (pan-Cytokeratin), stromal fibroblasts (Vimentin) and vascular smooth muscle (α-SMA); a complex mammary adenoma was used as a positive control for Calponin. Within the tumor, the tubular cells had strong and diffuse cytoplasmic staining with anti-Cytokeratin antibody (Fig. 3), while the stromal-like component was had weak and patch positivity. Calponin stain was diffusely and strongly positive in the stromal-like component, thus identified as myoepithelium, and completely negative in the tubular component (Fig. 4). Vimentin had a similar strong and diffuse immunoreactivity, while Alpha-SMA immunostaining was patchy and weak. Further images if IHC stains are given in Supplementary figure 5.

Histological diagnosis was tubular carcinoma and malignant myoepithelioma of apocrine sweat gland origin.

The day after surgery the dog was discharged from hospital with antibiotic and anti-inflammatory therapy.

At 10 day later, skin stitches were removed and another Ca concentration exam was performed with normal result (8.8 mg/dl RR: 8.4 to 11. mg/dl).

An 18-months-follow-up was obtained from the owners, confirming that the patient is healthy with no sign of local.

recurrence; an ultrasound abdominal evaluation did not find any sign of metastatic disease.

## Discussion and conclusion

Malignant neoplasms of apocrine sweat glands in general occur uncommonly in dogs and cats. The metastatic rate of these tumors is largely unknown, due to scarcity of reports; however, the biological behavior of apocrine cutaneous tumors may parallel the modified apocrine mammary gland, ranging from benign to aggressive, depending on the histological features (Gross et al. 2005). Complex and mixed tumors, that are common in canine mammary glands, are uncommon to rare in cutaneous apocrine sweat glands, and rarer are the tumors in which the myoepithelial component shows features of malignancy. Although in the past the definition of malignant mixed sweat gland tumors or carcinosarcomas has been proposed (Gross et al. 2005), a more precise definition of carcinoma and malignant myoepithelioma is preferred for those tumors in which both the epithelial and myoepithelial cells have features of malignancy (Goldschmidt et al. 2017). In the present case the criteria of malignancy were marked atypia and high mitotic rate in both Cytokeratin-positive epithelial and Calponin-positive myoepithelial components of the tumor.

Biological behavior of malignant sweat gland carcinoma and malignant myoepithelioma is still little studied in veterinary medicine, as a result of scarcity of reports. A case of “mixed apocrine gland tumor” is described by Morita et al. (2010) in a dog with bone and bone marrow metastases; interestingly, also in this case the tumor included a “true” epithelial component and a second myoepithelial population, that was immunostained by Calponin and Vimentin; unfortunately, in this case no details are given about clinical and clinic-pathological data (Morita et al. 2010).

The role of myoepithelial cells in apocrine carcinoma remains an area of active investigation; paralleling to mammary tumors, they could play a “protective” role by secreting angiogenic inhibitors and tumor suppressor proteins, but the precise influence on tumor behavior probably depends on the degree of differentiation of myoepithelial cells (Sánchez-Céspedes et al. 2016). Shiraki et al. (2012) reported a unique case of “apocrine carcinoma with dominant myoepithelial proliferation” similar to the case here reported, suggesting that the behavior of myoepithelial cells can significantly influence tumor aggressiveness and outcome. Recently, Matsumoto et al. (2023), described a case of canine apocrine carcinoma and malignant myoepitehlioma, where the malignant progression involved contemporaneously the luminal epithelium and myoepithelium. Interestingly, despite the high-grade histological features, metastases didn’t occur, as in our case. The myoepithelial component in canine mammary tumors seems to have a rather protective effect on metastatic rate as carcinoma and malignant myoepithelioma has a less aggressive biological behavior, with lower metastatic rate, when compared with simple carcinomas (Shiraki et al. 2012; Mastumoto et al., 2023). Further studies and reports are needed to address the prognosis of apocrine sweat gland carcinoma and malignant myoepitelioma in dogs.

Differential diagnoses for hypercalcemia include primary hyperparathyroidism, other neoplastic diseases, secondary hyperparathyroidism, vitamin D toxicosis, hypoadrenocorticism, acute renal failure, and granulomatous disease. In the present case, these conditions were excluded based on the physical examination, diagnostic imaging, and clinical pathology results obtained during the initial investigation. Hypoadrenocorticism has been considered unlikely because Na/K ratio was 28,84; the normal Na/K ratio is between 27 and 40; 95% of dogs with hypoadrenocorticism has ratios 27 or lower. (Klein 2010). A basal cortisol concentration could have more definitively excluded hypoadrenocorticism as it has a high negative predictive value. Primary hyperparathyroidism could have been more definitively excluded by measuring the serum PTH concentration, which in that disease would be elevated despite the hypercalcemia. This was not performed in this case, as parathyroid nodules were not detected on ultrasound of the neck. Parathyroid nodules in dogs with primary hyperparathyroidism are usually visible due to their marked hypoechoic or anechoic appearance compared with the thyroid gland, and may measure about 2 mm (Wisner et al. 1993). Serum phosphorus concentrations are usually decreased with primary hyperparathyroidism, whereas the phosphorus concentration was normal in this dog at initial presentation.

In this case, hypercalcemia could be related to production of PTHrp by the tumor; unfortunately, serum PTHrp dosage was, at the moment, unavailable. An alternative way to demonstrate production of PTHrp by the tumor could be immunohistochemistry, that has been applied on normal canine tissues and different cancers, including normal and neoplastic canine mammary gland (Konno et al. 2000); unfortunately, immunohistochemical staining for PTHrp is not directly correlated to the development of hypercalcemia, as many patients with PTHrp-immunopositive carcinomas of anal sac glands were normocalcemic (Gröne and Werkmeis, 1994).

The main limitation of this report is the lack of dosage of ionized Calcium, that is generally assumed to be closely related to total calcium levels. Actually, the values have to be considered together with total proteins and albumin values, that were normal in this case, as part of the total Calcium is linked to protein, and a decrease of serum albumin can reduce this fraction of total Calcium. In 1633 canine serum samples, the diagnostic disagreement between serum iCa and tCa was 27%, and in dogs with chronic renal failure, this disagreement was 36%. In dogs, tCa measurement often fail to detect cases of hypocalcemia, overestimating normocalcemia; thus, low tCa is likely to represent a true hypocalcemia (Schenck and Chew 2008); on the contrary, in the study by Groth et al. (2018) even a slightly increased tCa concentration (> 12.0 mg/dL) was shown to be highly predictive of ionized hypercalcemia in adult dogs without hyperphosphatemia, as in our case; actually, there is no optimal threshold for patients with hyperphosphatemia. Ionized hypercalcemia is rare in dogs without increased tCa concentrations, regardless of phosphorus status (Groth et al. 2018).

Given the exclusion of other cause of hypercalcemia, and the fact that hypercalcemia resolved after surgical excision of the tumor, the cutaneous tumor has been regarded as the cause of hypercalcemia in this dog. To the best of the author’s knowledge, this is the first report of hypercalcemia due to cutaneous apocrine sweat gland tumor.

Thus, cutaneous apocrine sweat gland tumors should be considered, aside of other tumors derived from modified apocrine glands (anal sac gland carcinomas and, to a lesser extent, mammary carcinomas), in the list of differentials in a dog with hypercalcemia of malignancy.

## Supplementary Information

Below is the link to the electronic supplementary material.

Supplementary figure 1 (JPG 461 KB)

Supplementary figure 2 (JPG 362 KB)

Supplementary figure 3 (PNG 4.22 MB)

High resolution image (TIFF 8.71 MB)

Supplementary figure 4 (PNG 4.52 MB)

High resolution image (TIFF 9.04 MB MB)

Supplementary figure 5 (PNG 5.04 MB)

High resolution image (TIFF 11.3 MB)

Supplementary Material 6 (PDF 80.9 KB)

## Data Availability

No data has been created during the current study.
