# Supplementary figures and images for: Hypercalcemia of malignancy in a dog with cutaneous apocrine gland carcinoma and malignant myoepithelioma
Source: Vet Res Commun. 2026 Jan 20;50(2):114. doi: 10.1007/s11259-025-11011-4 (PMC12819430; doi:10.1007/s11259-025-11011-4)

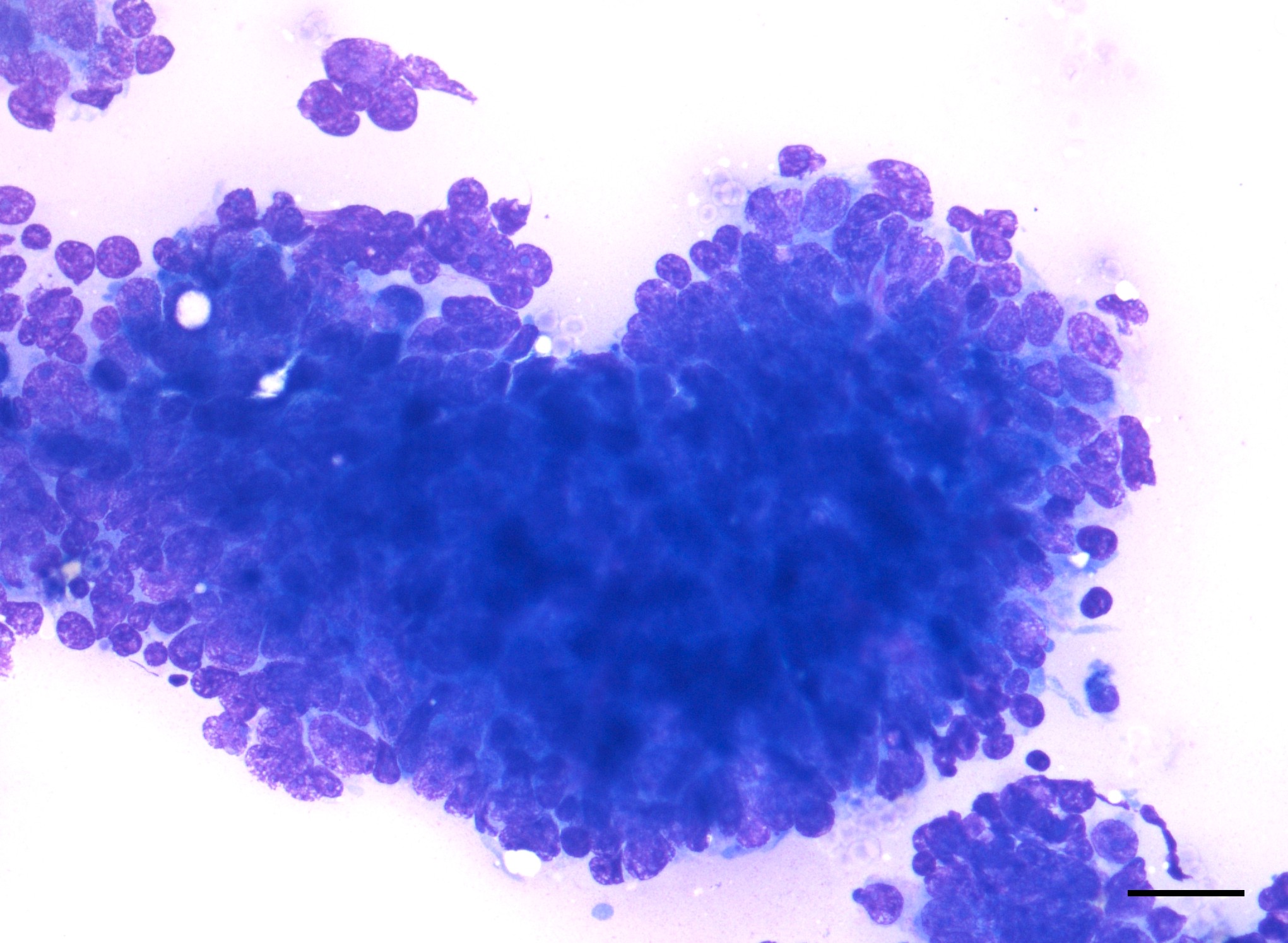

Supplement: Supplementary file 1 — (JPG 461 KB) [file 11259_2025_11011_MOESM1_ESM.jpg]

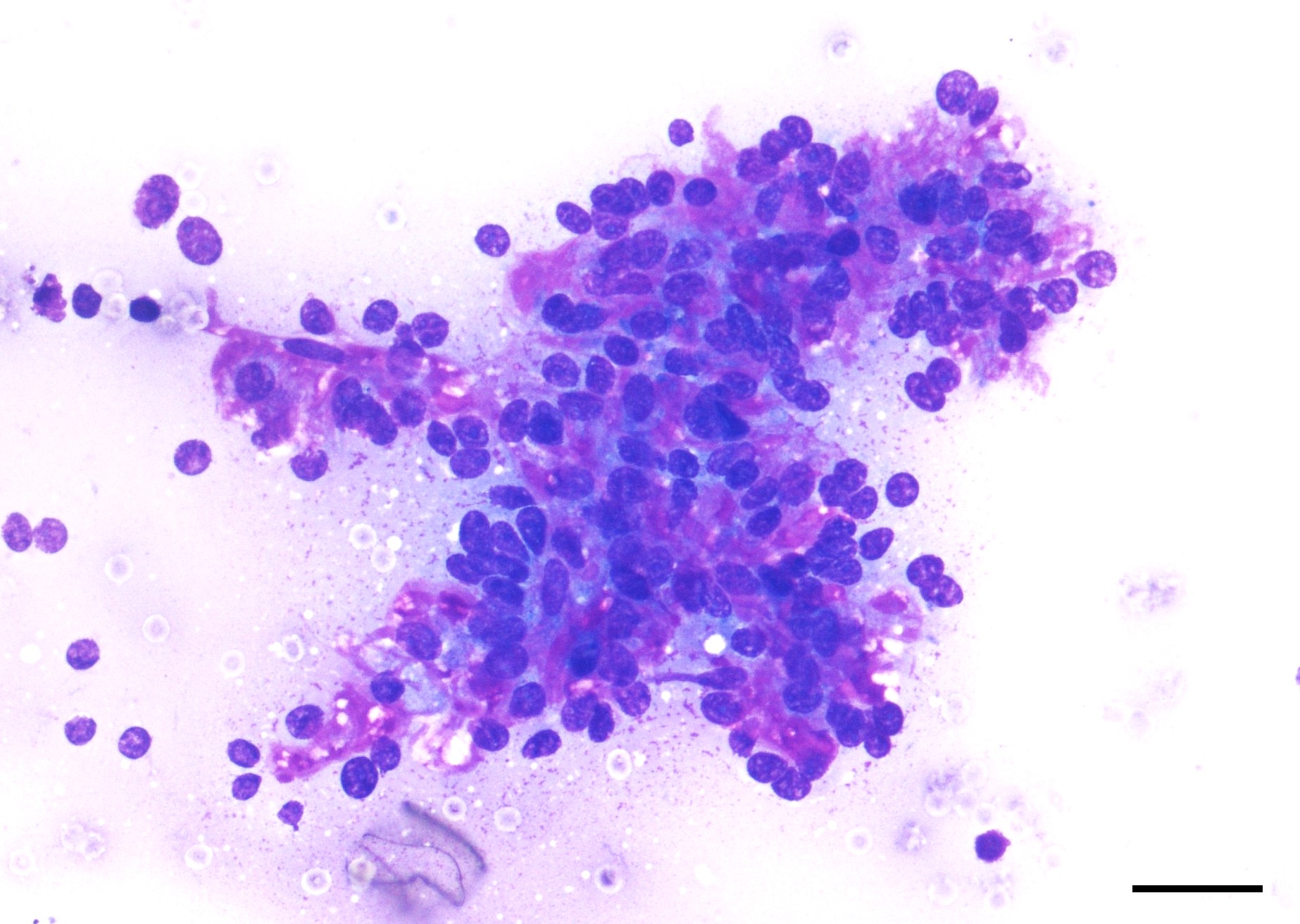

Supplement: Supplementary file 2 — (JPG 362 KB) [file 11259_2025_11011_MOESM2_ESM.jpg]

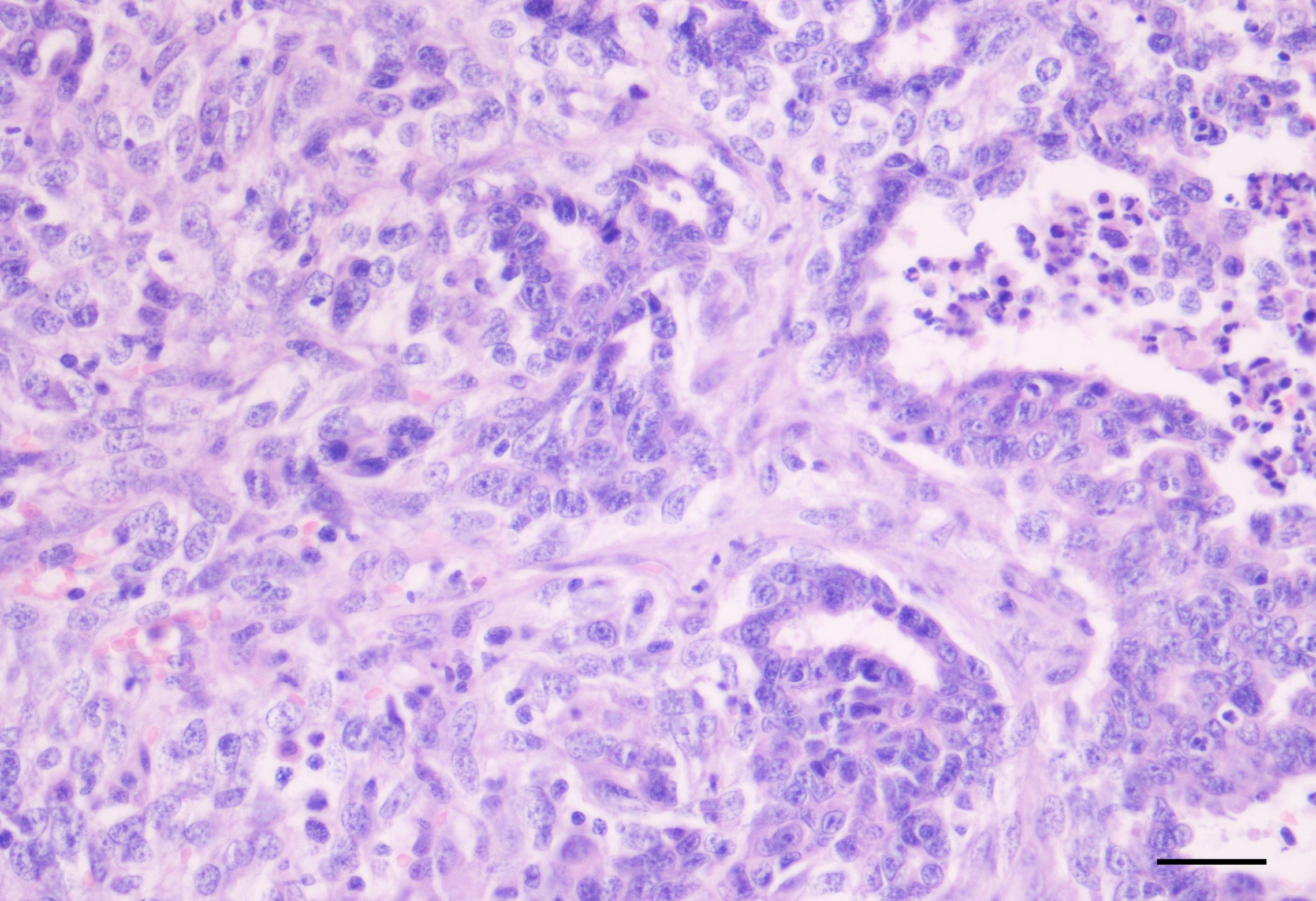

Supplement: Supplementary file 3 — (PNG 4.22 MB) [file 11259_2025_11011_Fig5_ESM.png]

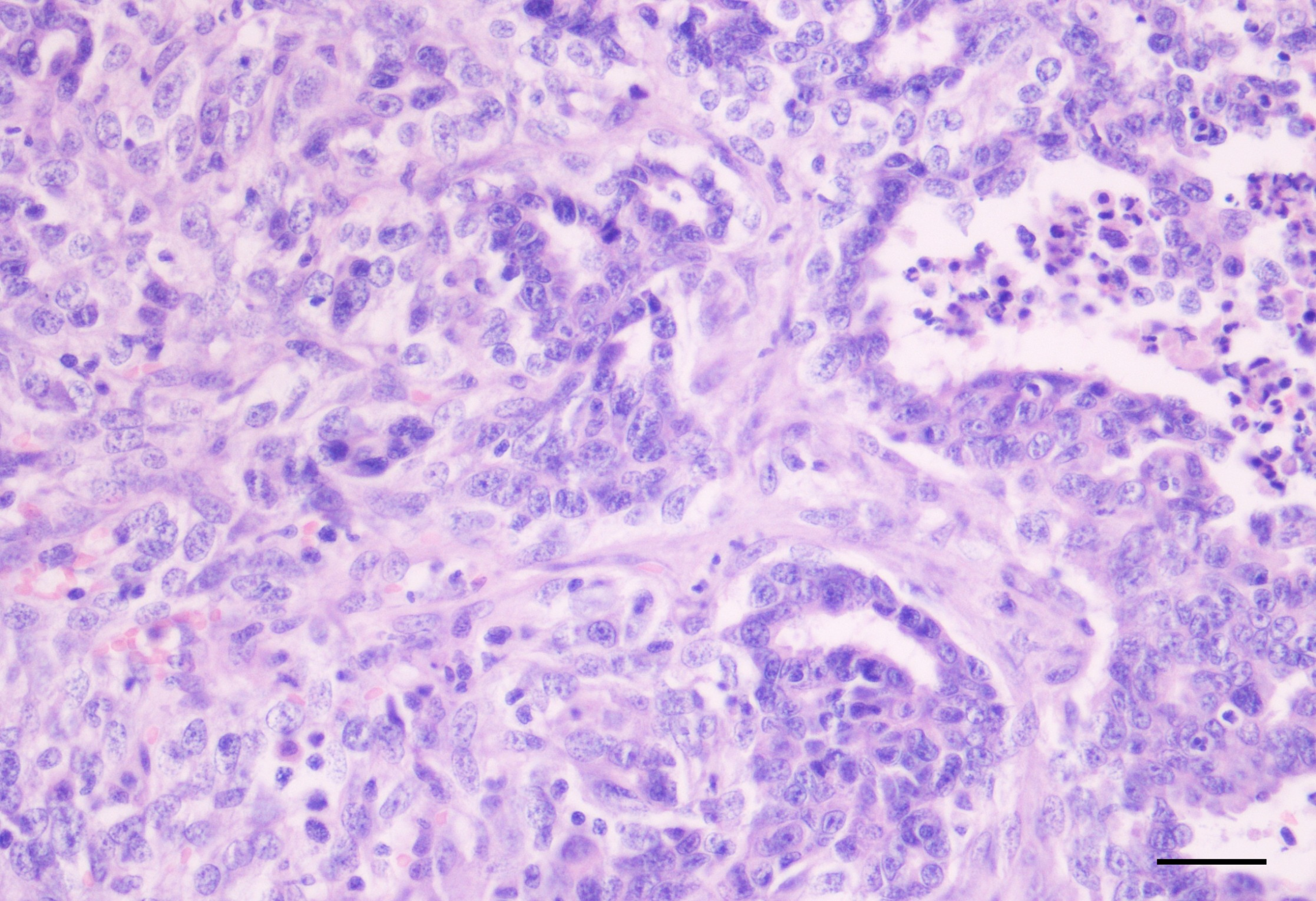

Supplement: Supplementary file 4 — High resolution image (TIFF 8.71 MB) [file 11259_2025_11011_MOESM3_ESM.tiff]

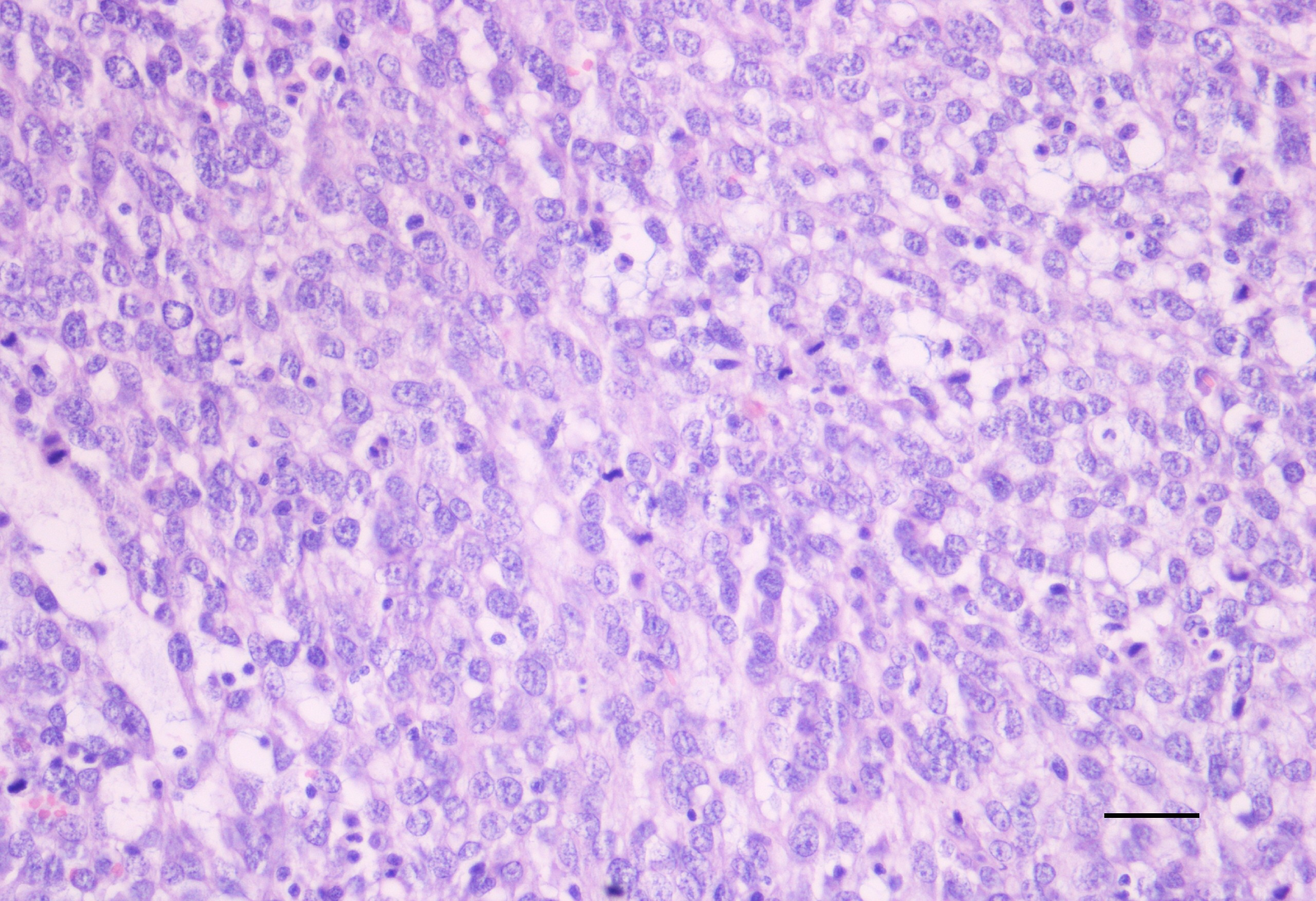

Supplement: Supplementary file 5 — (PNG 4.52 MB) [file 11259_2025_11011_Fig6_ESM.png]

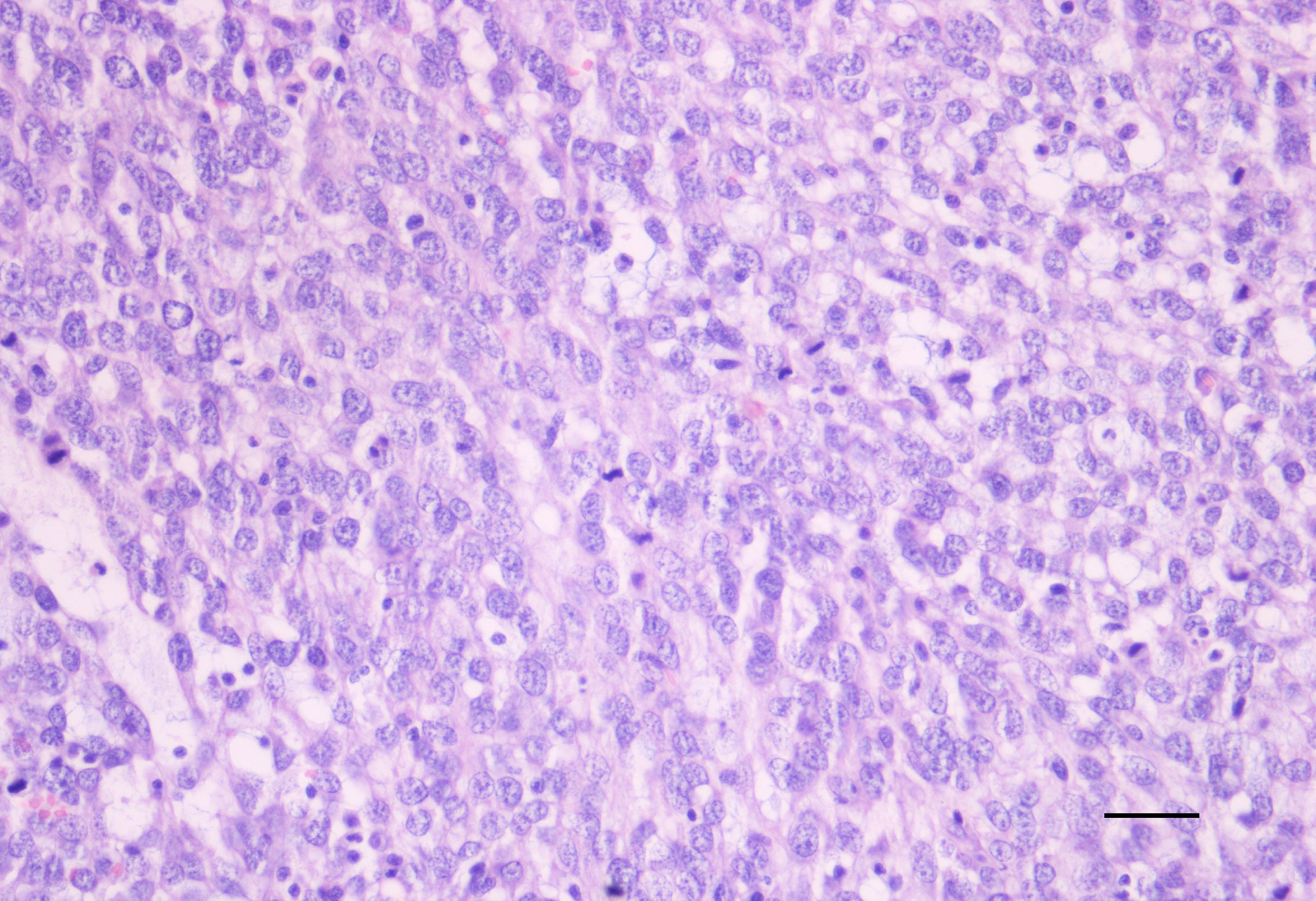

Supplement: Supplementary file 6 — High resolution image (TIFF 9.04 MB MB) [file 11259_2025_11011_MOESM4_ESM.tiff]

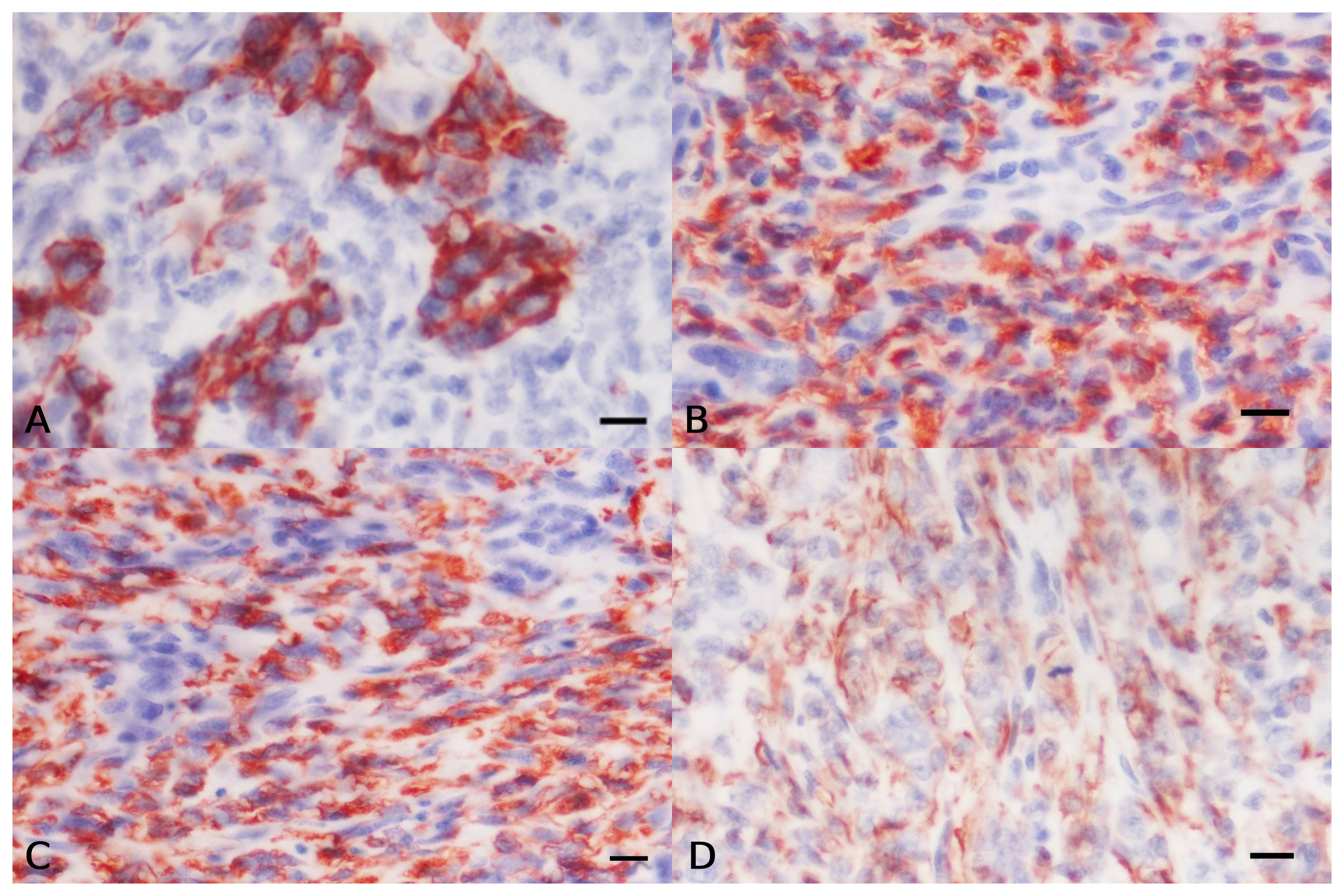

Supplement: Supplementary file 7 — (PNG 5.04 MB) [file 11259_2025_11011_Fig7_ESM.png]

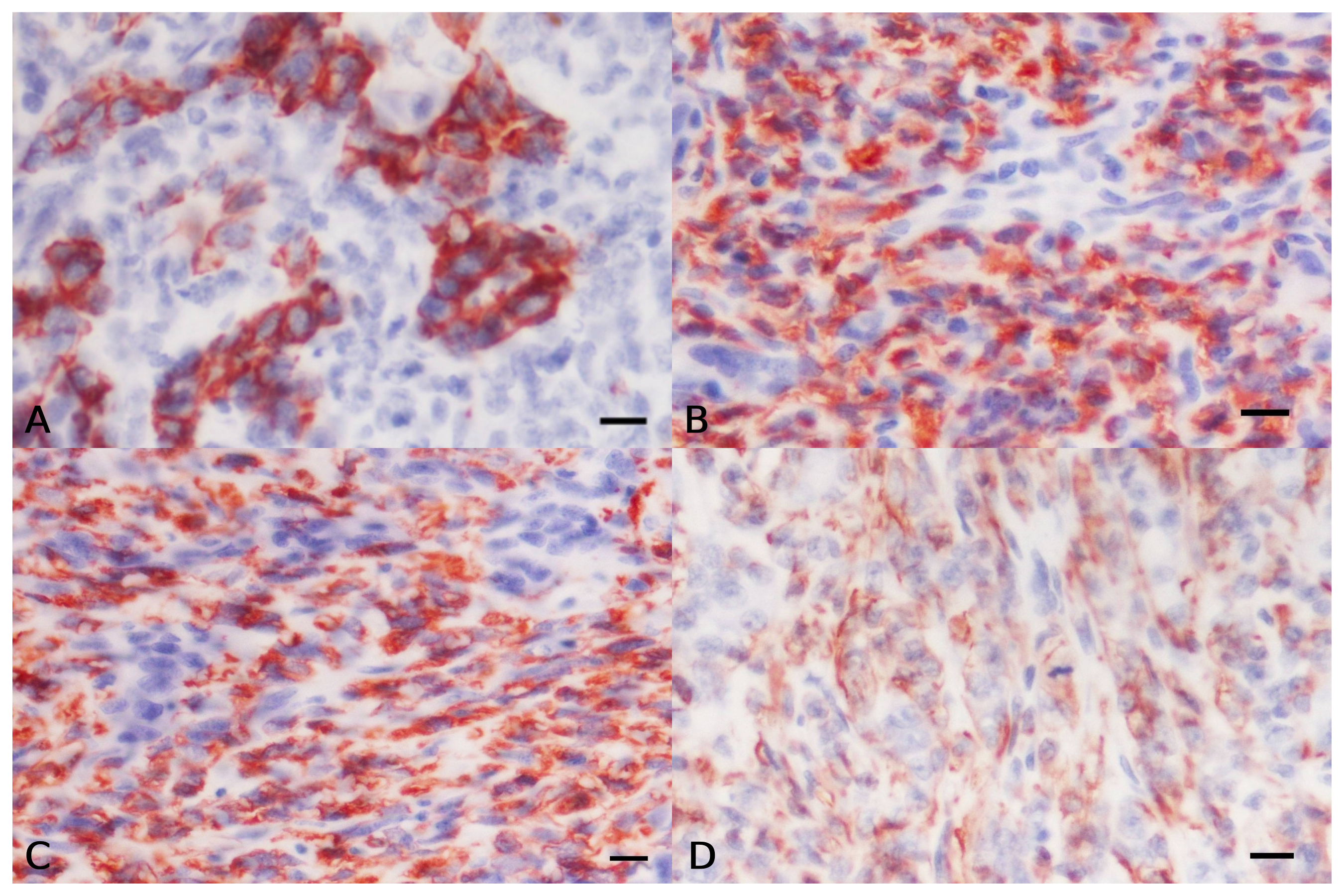

Supplement: Supplementary file 8 — High resolution image (TIFF 11.3 MB) [file 11259_2025_11011_MOESM5_ESM.tiff]
