# Supplementary material for: Hypercalcemia of malignancy in a dog with cutaneous apocrine gland carcinoma and malignant myoepithelioma
Source: Vet Res Commun. 2026 Jan 20;50(2):114. doi: 10.1007/s11259-025-11011-4 (PMC12819430; doi:10.1007/s11259-025-11011-4)
Supplement: Supplementary file 9 — (PDF 80.9 KB) [file 11259_2025_11011_MOESM6_ESM.pdf]

SUPPLEMENTAL MATERIAL FROM

Title

Hypercalcemia of malignancy in a dog with cutaneous apocrine gland carcinoma and malignant myoepithelioma.

Full author names

Ludovica Emiliani Pescitelli[1], Ambra Luisa Misia[2], Giulia Moretti[2], Giovanni Angeli[2], Maria Teresa Antognoni[2], Antonello Bufalari[2], Eleonora Scorsi[2,3], Elvio Lepri[2✢].

Full institutional mailing addresses

[1]Private practitioner, Veterinary Clinic Giaconella, Via Eugenio Checchi 57, 00157, Rome, Italy

[2] Department of Veterinary Medicine, University of Perugia, Via San Costanzo, 4, 06126, Perugia, Italy

[3] CDvet Research, Via giovanni nicotera, 7, 00195 – Roma, Italy

✢e-mail: elvio.lepri@unipg.it

Online Resource 1. Skin, dog. Large three-dimensional cluster of strictly packet cells with hyperchromatic cytoplasm and nucleus, in columnar to acinar arrangement. May-Grünwald-Giemsa, bar 25 μm

Online Resource. Skin, dog. Loosely arranged cells with poorly distinct cell borders and pale cytoplasm, embedded in a bright pink extracellular material. May-Grünwald-Giemsa, bar 25 μm

Online Resource 3. Skin, dog. Irregular tubules lined by cuboidal cells with marked anisokaryosis and multiple nucleoli. HE, bar 25 μm.

Online Resource 4. Skin, dog. Polygonal to spindle cells embedded in loose stroma, showing high mitotic activity. Several infiltrating lymphocytes are present. HE, bar 25 μm.

Online Resource 5. Skin, dog. Higher magnification of IHC results. A: anti-panCytokeratin; B: anti-Vimentin; C: anti-Calponin; D: anti-α-SMA. Aminoethyl carbazole / Hematoxylin, bar 10 μm.
